# Association between Polymorphisms and Haplotype in the ABCA1 Gene and Overweight/Obesity Patients in the Uyghur Population of China

**DOI:** 10.3390/ijerph13020220

**Published:** 2016-02-15

**Authors:** Ming-Hong Yao, Jia He, Ru-Lin Ma, Yu-Song Ding, Heng Guo, Yi-Zhong Yan, Jing-Yu Zhang, Jia-Ming Liu, Mei Zhang, Dong-Shen Rui, Qiang Niu, Shu-Xia Guo

**Affiliations:** Department of Public Health and Key Laboratory of Xinjiang Endemic and Ethnic Diseases of the Ministry of Education, Shihezi University School of Medicine, Shihezi 832002, China; ymhldjxa@sina.com (M.-H.Y.); hejia123.shihezi@163.com (J.H.); marulin@126.com (R.-L.M.); 13399931625@163.com (Y.-S.D.); guoheng@shzu.edu.cn (H.G.); erniu19880215@sina.com (Y.-Z.Y.); yfyxxzjy@126.com (J.-Y.Z.); liujiaming@shzu.edu.cn (J.-M.L.); zmberry@foxmail.com (M.Z.); ruidongsheng@gmail.com (D.-S.R.); niuqiang1214@163.com (Q.N.)

**Keywords:** Uyghur, ABCA1, polymorphism, haplotype, overweight/obese

## Abstract

*Objective*: This study aimed to detect the association between polymorphisms and haplotype in the ATP-binding cassette transporter A1 (ABCA1) gene and overweight/obese Uyghur patients in China. *Methods*: A total of 259 overweight/obese patients and 276 normal weight subjects, which were randomly selected from among 3049 adult Uyghurs, were matched for age. We genotyped ABCA1 single nucleotide polymorphisms of rs2515602, rs3890182, rs2275542, rs2230806, rs1800976, and rs4149313. *Results*: (1) The genotypic and allelic frequencies of rs2515602 and rs4149313 differed between the control group and case group. The genotypic frequency of rs2275542 also differed between the control group and case group (*p* < 0.05); (2) rs2515602, rs2230806, and rs4149313 polymorphisms were significantly related to risk of overweight/obese; (3) a significant linkage disequilibrium (LD) was observed between the ABCA1 gene rs2275542 with rs3890182 and rs2515602 with rs4149313. (4) the C-C-C-A-G-G, T-C-G-A-G-G, and T-T-G-G-G-A haplotypes were significant in normal weight and overweight/obese subjects (*p* < 0.05); (5) the levels of HDL-C (rs2515602, rs2275542, rs4149313) in normal weight subjects were different among the genotypes (*p* < 0.05); the levels of TC, LDL-C and TG (rs1800976) in overweight/obese subjects were different among the genotypes (*p* < 0.05). *Conclusions:* The rs2515602, rs4149313, and rs2275542 polymorphisms were associated with overweight/obese conditions among Uyghurs. Strong LD was noted between rs2275542 with rs3890182 and rs2515602 with rs4149313. The C-C-C-A-G-G and T-C-G-A-G-G haplotypes may serve as risk factors of overweight/obesity among Uyghurs. The T-T-G-G-G-A haplotype may serve as a protective factor of overweight/obesity among Uyghurs. Rs2515602, rs2275542, rs4149313, and rs1800976 polymorphisms in the ABCA1 gene may influence lipid profiles.

## 1. Introduction

The ATP-binding cassette transporter A1 (ABCA1) gene, which is located on chromosome 9q31, and consists of 49 exons, encodes the key protein that effects the efflux of excess lipids from the peripheral cells into lipid-poor apolipoprotein A1 particles, and facilitates the formation of high-density lipoprotein-cholesterol (HDL-C) [1,2]. ABCA1 gene mutations may affect the transcription and expression of the protein, thereby affecting serum lipid levels [3,4]. Numerous studies have focused on the association of ABCA1 gene polymorphism, serum lipid levels, and coronary heart disease, however, few studies have examined the relationship between ABCA1 gene polymorphisms and other diseases. In recent years, increased understanding of the expression pattern of ABCA1 in various tissues has led to the suggestion that ABCA1 plays a role beyond HDL-C metabolism [5,6]. In this regard, Le *et al.*’s [7] studies showed that an increase in ABCA1 expression induces the differentiation of 3T3-L1 preadipocytes into mature adipocytes. The preceding observations indicate that ABCA1 gene polymorphisms and overweight/obesity are correlated. The Uyghur nationality in Xinjiang has a unique culture and customs, and the prevalence of overweight/obesity is higher in this nationality than in the Han nationality living in the same area [8]. Whether ABCA1 gene polymorphisms and overweight/obesity are correlated among Uyghurs should be determined. This study will help clarify the important risk factors of overweight/obese in the Uyghur nationality. Accordingly, we investigated six (rs2515602, rs3890182, rs2275542, rs2230806, rs1800976 and rs4149313) single nucleotide polymorphisms (SNPs) in the ABCA1 gene in a sample consisting of 535 (normal weight: 276 and overweight/obese: 259) individuals to determine the association genetic variations in the ABCA1 gene and overweight/obese among Uyghurs. In addition, we also discussed the relationship between ABCA1 gene variant and serum lipids. The SNPs were selected according to the previous findings of other studies [9,10,11]. A case-control study was adopted to detect the association of six SNPs, haplotypes and linkage disequilibrium (LD) in ABCA1 gene and overweight/obese as well as serum lipids among overweight/obese Uyghur patients.

## 2. Materials and Methods

The protocol was approved by the Institutional Ethics Review Board (IERB) of the First Affiliated Hospital of Shihezi University School of Medicine (IERB No. SHZ2010LL01). Written informed consent was obtained from each participant. Standard university hospital guidelines, including informed consent, confidentiality, voluntary participation, and anonymity were followed. All participants gave written informed consent before the study began.

### 2.1. Study Population

The total subjects in this study consisted of 535 unrelated adults who reside in Jiashi County, Xinjiang Uyghur Autonomous Region, People’s Republic of China. They were randomly selected from our previous stratified randomized cluster samples [8]. A total of 259 overweight/obese patients were randomly selected as the case group, and 276 normal weight subjects were randomly selected as the control group using the group-matching method.

### 2.2. Epidemiological Survey and Biochemical Measurements

Information on demographic and personal lifestyles was collected with a self-developed questionnaire during face-to-face interviews. Blood pressure, height, weight, waist circumference, and hip circumference were measured according to standardized methods [12]. Body mass index (BMI) was calculated by weight (kg) divided by the square of the measured height (m^2^). After overnight fasting, venous blood samples (5 mL) were drawn from the forearm vein of all participants. A part of the blood (3 mL) was collected into glass tubes and used to determine the serum lipid levels. Another part of the blood (2 mL) was transferred into tubes and used to extract DNA. The concentrations of triglycerides (TGs), total cholesterol (TC), HDL-C, and low -density lipoprotein-cholesterol (LDL-C) in serum were measured with a DXC-800 automatic biochemical analyzer (Beckman, Pasadena, CA, USA) at the Clinical Science Experiment Center of the First Affiliated Hospital of Shihezi University School of Medicine.

### 2.3. Diagnostic Criteria

The diagnostic criteria of overweight, obesity and normal weight were defined as a BMI 24–28, >28, and <24 kg/m^2^; respectively [13]. Low HDL-C, high TC, high LDL-C, and high TG were defined as HDL-C < 1.03 mmol/L, TC > 5.17 mmol/L, LDL-C > 2.59 mmol/L, TG > 1.70 mmol/L, respectively [14]. Hypertension was defined as systolic blood pressure ≥ 140 mmHg and/or diastolic blood pressure ≥ 90 mmHg.

### 2.4. DNA Extraction

Fasting venous blood (200 µL) was taken from each study subject, and a blood genomic DNA isolation kit (non-centrifugal column, TIANGEN, Beijing, China) was used to extract the whole blood genomic DNA. The extracted DNA was verified by gel electrophoresis (0.7% agarose). A NanoDrop spectrophotometer (Thermo Scientific, Waltham, MA, USA) was used for quantitative determination of DNA concentration and purity: concentration ≥30 ng/µL and purity levels (OD_260_/OD_280_) of 1.7–2.0 were considered acceptable. Samples that met these criteria were diluted to 10–30 ng/µL with double-distilled water and then stored at −80 °C.

### 2.5. PCR Amplification

The extracted DNA was stored at 4 °C until analysis. Genotyping of ABCA1 gene rs2515602, rs3890182, rs2275542, rs2230806, rs1800976, and rs4149313 were performed via the SNaPshot technique. The sequences of the forward and reverse primers used for the genotyping of six single SNP are list in Table 1. Final PCR reaction volumes were 15 µL, which included 1 µL DNA samples, 0.3 µL dNTPs, 7.4 µL water, 1.5 μL 10 × PCR buffer, 1.5 µL MgCl_2_, 0.3 µL Taq enzymes, and 3 μL mixture of PCR amplification primers. The cycling conditions were as follows: predegeneration at 94 °C for 4 min; followed by 35 cycles of denaturation at 94 °C for 20 s, annealing at 56 °C for 30 s, and extension at 72 °C for 1 min. A final extension step was conducted at 72 °C for 3 min, after which samples were maintained at 4 °C. The reactions were set up in an ice bath, and each PCR experiment included a negative control reaction.

**Table 1:** Sequences of forward and reverse primers to genotype the ABCA1 gene.

SNPs	Forward Sequence	Reverse Sequence	PCR Product	Allele
rs2515602	5′-CAGTGAAAACAATGGTGAGGC-3′	5′-CATCTATGTGGAGAGATGTGG-3′	235bp	A/G
rs3890182	5′-AAGAACACTCGCAAAGTCAGC-3′	5′-TGTGTTTTTCAGGTGCCCTTG-3′	208bp	C/T
rs2275542	5′-AATGCAGTTGGCAGCAATCTG-3′	5′-TCCCATTAGATCTTCCCCAAG-3′	208bp	A/G
rs2230806	5′-CTTGTGCTTGTCTCTCTTTGC-3′	5′-ATTGGCTTCAGGATGTCCATG-3′	237bp	C/G
rs1800976	5′-GGAACGTGGACTAGAGAGTCTG-3′	5′-AGTCACTCAGCAGAAAGCACG-3′	216bp	C/T
rs4149313	5′-TGGGAAACCCTCAGAATACTG-3′	5′-GTTAGCAGAGGCAGCAGCACTA-3′	210bp	A/G

### 2.6. PCR Products Purification

Shrimp alkaline phosphatase (SAP) was used to remove excess dNTPs from samples after PCR. This step served to ensure the accuracy of single-base extension. The final SAP reaction volumes were 5.0 mL, which included 0.5 µL 10 × SAP buffer, 2 uL of PCR product, 2 µL of double-distilled water, and 0.5 µL of SAP enzyme. The reactions were conducted by incubation at 37 °C for 40 min, followed by incubation at 85 °C for 5 min. The reaction products were stored at 4 °C.

### 2.7. Single-Base Extension

For single-base extension reactions, the final reaction volumes were 6.0 µL, which included 0.5 μL of SNaPshot reagent, 2.5 µL of water, 1 µL of primer mix, and 2 uL of purified PCR products. The reaction conditions were as follows: denaturation at 94 °C for 30 s, followed by 40 cycles of 94 °C for 5 s, 52 °C for 5 s, and finally 52 °C for 5 s. The reaction products were stored at 4 °C.

### 2.8. Genotyping Analysis

Genotyping analysis was as follows: about 1 µL of reaction product and 9 µL HIDI were obtained. Denaturation was conducted at 95 °C for 3 min. Samples were then immediately chilled in an ice-water bath. All representative SNP genotyping experiments were performed via TaqMan technology on an ABI3730XL system (Applied Biosystems, Foster City, CA, USA). GeneMapper was used to complete the classification and output the results.

### 2.9. Statistical Analysis

Epidata 3.02 software was used to establish a database, and the double entry method was used for data input and logic error detection. Non-normally distributed continuous variables such as TG, TC and LDL-C are shown as median and interquartile range (25th, 75th percentile), while HDL-C concentrations, age, height, weight, BMI, waist circumference, hip circumference, waist-to-hip ratio, systolic blood pressure, diastolic blood pressure and Pulse pressure are presented as mean ± standard deviation. The frequency of the ABCA1 alleles was determined by gene counting. Chi-square tests were used to compare the differences in percentages and to assess Hardy-Weinberg expectations. The Kruskal-Wallis H statistic or One Way ANOVA were used to compare continuous variables among the three genotype groups, while the Kolmogorov-Smirnov Z test or the Student’s *t*-Test. Single factor logistic regression analysis was used to assess risk factors of overweight/obesity (overweight/obesity as the dependent variable (0 = no, 1 = yes)), independent variable included sex (1 = male, 2 = female), age (1 = 18–30, 2 = 31–40, 3 = 41–50, 4 = 50–60, 5 = 61~), hypertension (0 = no, 1 = yes), high TC (0 = no, 1 = yes), high TG (0 = no, 1 = yes ), high LDL-C (0 = no, 1 = yes), smoker (0 = no, 1 = yes), drinker (0 = no, 1 = yes), rs2515602 (CC = 1, CT = 2, TT = 3), rs3890182 (GG = 1, AG = 2, AA = 3), rs2275542 (CC = 1, CT = 2, TT = 3), rs2230806 (AA = 1, AG = 2, GG = 3), rs1800976 (CC = 1, CG = 2, GG = 3), rs4149313 (AA = 1, AG = 2, GG = 3). Frequency table and statistical analysis were used with SPSS 17.0 (SPSS Inc., Chicago, IL, USA) statistical package. SHEsis software was used to analysis haplotype and LD [15]. A *p* value of 0.05 was used to define the level of significance.

## 3. Results

### 3.1. Clinical Data and Biochemical Characteristics of Study Subjects

Table 2 shows the clinical profiles of the participants. A low male-to-female ratio, weight, BMI, waist circumference, hip circumference, waist-to-hip ratio, systolic blood pressure, diastolic blood pressure, pulse pressure, TC, LDL-C, and TG were recorded in all normal weight compared with overweight/obese subjects (*p* < 0.05). The levels of serum HDL-C were higher in overweight/obese subjects than those in normal weight subjects (*p* < 0.001). No significant differences were found in the values of average age, smokers, and drinkers between overweight/obese and normal weight subjects (*p* > 0.05).

**Table 2:** General characteristics and serum lipid levels between the control group and case group.

Characteristics	Control (*n* = 276)	Case (*n* = 259)	*t* (χ^2^, Z)	*p*
Male/female	124/152	144/115	6.086	0.014
Age, years	44.32 ± 16.04	43.95 ± 14.11	0.282	0.778
Height, cm	159.25 ± 8.53	159.59 ± 8.44	−0.459	0.646
Weight, kg	52.99 ± 6.97	69.19 ± 9.82	−22.105	*p* < 0.001
BMI, kg/m^2^	20.81 ± 1.29	27.10 ± 2.69	−34.776	*p* < 0.001
Waist circumference, cm	80.64 ± 7.35	92.84 ± 10.43	−15.711	*p* < 0.001
Hip circumference	92.50 ± 5.47	101.05 ± 7.79	−14.763	*p* < 0.001
Waist-to-hip ratio	0.87 ± 0.06	0.92 ± 0.07	−8.235	*p* < 0.001
Systolic blood pressure, mmHg	125.21 ± 20.06	132.12 ± 21.37	−3.860	*p* < 0.001
Diastolic blood pressure, mmHg	78.42 ± 12.60	82.46 ± 13.84	−3.533	*p* < 0.001
Pulse pressure, mmHg	46.79 ± 14.36	49.66 ± 15.55	−2.222	0.027
TC, mmol/L	4.28 (3.73–4.88)	4.61 (4.02–5.43)	−4.103	*p* < 0.001
TG, mmol/L	0.91 (0.65–1.68)	1.83 (0.98–2.67)	−7.936	*p* < 0.001
HDL-C, mmol/L	1.22 ± 0.29	1.04 ± 0.29	7.283	*p* < 0.001
LDL-C, mmol/L	2.31 (1.84–2.86)	2.60 (2.04–3.08)	−3.678	*p* < 0.001
Smoker, *n* (%)	36 (13.04)	31 (11.97)	0.141	0.708
Drinker, *n* (%)	5 (1.81)	10 (3.86)	2.509	0.151

Notes: BMI, Body mass index; TG, triglyceride; TC, total cholesterol; LDL-C, low-density lipoprotein-cholesterol; HDL-C, high-density lipoprotein-cholesterol.

### 3.2. Genotype and Allele Frequencies

The genotype and allele frequencies of the SNPs between normal weight and overweight/obese subjects are presented in Table 3. The genotypic frequencies of the six SNPs were all in Hardy-Weinberg equilibrium (*p* > 0.05). The genotypic and allelic frequencies of rs3890182, rs2230806 and rs1800976 between the control group and case group showed no significant differences (*p* > 0.05). For rs2515602 polymorphism, we observed a lower frequency of the T allele (37.1% *vs.* 46.7, *p* = 0.001) in overweight/obese patients compared with normal weight subjects. Rs2275542 genotypes were significance differences between normal weight and overweight/obese subjects (*p* = 0.033). Moreover, the frequency of rs4149313 G allele in case group was higher than in control group (51.9% *vs.* 44.4%, *p* = 0.014).

**Table 3:** The genotypic and allelic frequencies between the subjects with normal weight and overweight/obese (*n* (%)).

SNPs	Group	Genotype Distribution *n* (%)			*χ^2^*	*p*	HWE-P	MAF *n* (%)	*χ^2^*	*p*
		R/R	C/R	C/C						
rs2515602	control	70 (25.4)	118 (42.8)	88 (31.9)	14.69	0.001	0.231	R: 258 (46.7)	10.26	0.001
	case	32 (12.4)	128 (49.4)	99 (38.2)			0.751	R: 192 (37.1)		
rs3890182	control	260 (94.2)	14 (5.1)	2 (0.7)	2.746	0.253	0.462	R: 18 (3.3)	2.521	0.112
	case	250 (96.5)	9 (3.5)	-			0.999	R: 9 (1.7)		
rs2275542	control	41 (14.9)	119 (43.1)	116 (42.0)	6.803	0.033	0.765	R: 201 (36.4)	2.686	0.101
	case	20 (7.7)	124 (47.9)	115 (44.4)			0.462	R: 164 (31.7)		
rs2230806	control	40 (14.5)	117 (42.4)	119 (43.1)	4.334	0.115	0.654	R: 197 (35.7)	3.105	0.078
	case	42 (16.2)	128 (49.4)	89 (34.4)			0.936	R: 212 (40.9)		
rs1800976	control	50 (18.1)	127 (46.0)	99 (35.9)	0.42	0.811	0.870	R: 227 (41.1)	0.33	0.566
	case	49 (18.9)	124 (47.9)	86 (33.2)			0.966	R: 222 (42.9)		
rs4149313	control	55 (19.9)	135 (48.9)	86 (31.2)	6.257	0.044	0.985	R: 245 (44.4)	6.097	0.014
	case	74 (28.6)	121 (46.7)	64 (24.7)			0.780	C: 249 (48.1)		

Notes: R: rs2515602-T; rs3890182-G; rs2275542-T; rs2230806-A; rs1800976-C; rs4149313-G. C: rs2515602-C; rs3890182-A; rs2275542-C; rs2230806-G; rs1800976-G; rs4149313-A. HWE-P, Hardy-Weinberg equilibrium *p* value. MAF: minor allele frequencies.

### 3.3. Association of ABCA1 SNPs in Normal Weight and Overweight/Obese Individuals

The relationship of the six SNPs in ABCA1 and overweight/obese are summarized in Table 4. The risk degree of overweight/obese was evaluated by single factor logistic regression analysis after controlling for potential confounders which included sex, age, hypertension, high TC, high LDL-C, high TG, cigarette smoker, and alcohol drinker. The results show that rs2515602, rs2230806, and rs4149313 variants were independently associated with overweight/obese. Even if the results were corrected for multiple testing, rs2515602 and rs4149313 are still significant.

**Table 4:** The relationship between ABCA1 gene variant and overweight/obese.

SNPs	*β*	*SE*	*Waldχ2*	*p*	*OR*	*OR for 95% CI*
rs2515602	−0.481	0.14	11.792	0.001	0.618	0.470–0.814
rs2230806	−0.286	0.138	4.322	0.038	0.751	0.573–0.984
rs4149313	0.39	0.136	8.193	0.004	1.477	1.131–1.928
rs2275542	0.216	0.131	2.717	0.099	1.241	0.960–1.604
rs3890182	−0.036	0.137	0.069	0.792	0.965	0.738–1.261
rs1800976	0.027	0.123	0.048	0.826	1.027	0.808–1.307

Notes: β: regression coefficient; SE: Standard error; OR: odds ratio; CI: confidence interval.

### 3.4. Pairwise LD between SNPs

Pairwise LD performed for all six SNPs identified in normal weight and overweight/obese individuals is shown in Table 5. Six SNPs in the ABCA1 gene (rs2275542-rs2515602-rs1800976-rs2230806-rs3980182-rs4149313) were in LD with D’ ranging from 0.011 to 0.921 and r^2^ ranging from 0.000 to 0.889. We can found that a strong pairwise LD was present in the ABCA1 for the following SNPs pairs: rs2275542 with rs3890182 (D’ = 0.921, r^2^ = 0.889) and rs2515602 with rs4149313 (D’ = 0.785, r^2^ = 0.413).

**Table 5:** Pairwise LD between six SNPs in ABCA1 in the normal weight and overweight/obesity.

SNPs	rs2275542	rs2515602	rs1800976	rs2230806	rs3890182	rs4149313
rs2275542	-	0.07	0.011	0.02	0.921	0.016
rs2515602	0.002	-	0.067	0.832	0.392	0.785
rs1890976	0.000	0.004	-	0.079	0.112	0.019
rs2230806	0.000	0.031	0.005	-	0.044	0.462
rs3890182	0.889	0.005	0.000	0.000	-	0.471
rs4149313	0.000	0.413	0.000	0.143	0.005	-

Note: the upper triangle is D’ value and the lower triangle is r^2^ value.

### 3.5. Haplotype Analysis

The results of haplotype analysis of the six SNPs are shown in Table 6. The global haplotype frequencies were significantly different between control group and case group (*p* < 0.001). C-C-C-A-G-G and T-C-G-A-G-G haplotypes were significantly more frequent in the case group than in the control group, whereas the T-T-G-G-G-A haplotypes was less frequent in the overweight/obese group than in the normal weight group (*p* < 0.05).

**Table 6:** Estimated haplotype frequencies of six SNPs in ABCA1 between overweight/obese group and normal weight group.

Haplotype	Case, *n* (%)	Control, *n* (%)	χ^2^	*p*	OR (95% CI)
C-T-C-G-G-A	30 (11.4)	35 (12.6)			
C-C-C-A-G-G	29 (11.2)	14 (5.2)	5.378	0.020	2.511 (1.143–5.516)
C-C-G-G-G-G	24 (9.2)	19 (7)	1.219	0.270	1.531 (0.717–3.268)
C-C-G-A-G-G	21 (8.1)	31 (11.1)	0.288	0.592	0.821 (0.399–1.688)
T-C-G-A-G-G	16 (6.2)	5 (1.8)	6.274	0.012	3.879 (1.285–11.710)
T-C-G-G-G-G	15 (5.6)	10 (3.8)	1.631	0.202	1.818 (0.722–4.578)
C-C-G-A-G-A	11 (4.2)	9 (3.3)	0.604	0.437	1.481 (0.548–4.004)
T-T-C-G-G-A	10 (3.9)	13 (4.6)	0.021	0.885	0.932 (0.363–2.398)
T-C-C-G-G-G	9 (3.5)	9 (3.2)	0.134	0.715	1.212 (0.432–3.404)
C-C-G-G-G-A	8 (3.1)	4 (1.4)	1.901	0.168	2.424 (0.670–8.769)
T-C-C-A-G-G	6 (2.2)	14 (5.2)	1.491	0.222	0.519 (0.180–1.502)
T-T-G-G-G-A	5 (1.8)	22 (7.8)	5.985	0.015	0.275 (0.094–0.807)

Notes: The frequency of haplotype was below 0.03 not included in the table, and the risk assessment was not performed; global *p* < 0.001; C-T-C-G-G-A was used as a reference haplotype for obtaining the Odds Ratio calculations; haplotypes of six SNPs in the following order (left to right): rs2275542 (C > T), rs2515602 (T > C), rs1800976 (G > C), rs2230806 (G > A), rs3890182 (G > A), rs41493133 (A > G).

### 3.6. Correlation between Genotypes and Serum Lipid Profiles between the Subjects with Normal Weight and Overweight/Obese

Table 7 shows the correlation between genotypes and serum lipid profiles between the subjects with normal weight and overweight/obesity. There are only two subjects with AA genotype in control group according to rs3890182, so the levels of TC, TG, LDL-C, and HDL-C were not presented. Rs2515602 with CC genotype has higher HDL-C levels than in with TT genotype in normal subjects (*p* < 0.05); serum HDL-C levels was higher in control according to rs2275542 with CC/CT genotype than in TT genotype (*p* < 0.05); in cases, the levels of TG, TC, and LDL-C were higher according to rs1800976 with CG genotype than in GG/CC genotype (*p* < 0.05); serum HDL-C level was higher in control group according to rs4149313 with GG genotype than in AA genotype (*p* < 0.05).

**Table 7:** The genotypes of six SNPs and serum lipid levels (mmol/L) between the subjects with normal weight and overweight/obese.

SNPs	Group	Genotype	*n* (%)	TG	TC	LDL-C	HDL-C
rs2515602	Control	CC	88 (31.9)	0.90 (0.60–1.52)	4.28 (3.77–4.87)	2.25 (1.85–3.20)	1.28 ± 0.28 *
		CT	118 (42.8)	0.94 (0.67–1.87)	4.27 (3.76–4.96)	2.40 (1.90–2.88)	1.22 ± 0.28
		TT	70 (25.4)	0.86 (0.61–1.64)	4.29 (3.65–4.69)	2.19 (1.74–2.93)	1.16 ± 0.32 *
	Case	CC	99 (38.2)	1.82 (0.92–2.51)	4.62 (4.05–5.43)	2.40 (1.92–3.06)	1.04 ± 0.28
		CT	128 (49.4)	1.83 (1.06–2.69)	4.64 (4.03–5.53)	2.68 (2.06–3.16)	1.03 ± 0.28
		TT	32 (12.4)	1.87 (0.77–2.64)	4.44 (3.97–5.43)	2.53 (2.20–2.91)	1.06 ± 0.33 *
rs3890182	Control	GG	260 (94.2)	0.92 (0.65–1.74)	4.30 (3.73–4.90)	2.32 (1.84–2.88)	1.22 ± 0.29
		GA	14 (5.1)	0.82 (0.62–1.82)	3.98 (3.86–4.48)	2.16 (1.96–2.54)	1.31 ± 0.27
	Case	GG	250 (96.5)	1.82 (0.98–2.67)	4.63 (4.03–5.48)	2.61 (2.04–3.08)	1.04 ± 0.29
		GA	9 (3.5)	2.49 (1.00–2.66)	4.32 (3.26–5.20)	2.26 (1.61–2.99)	0.95 ± 0.20
rs2275542	Control	CC	116 (42.0)	0.86 (0.61–1.65)	4.29 (3.65–4.85)	2.31 (1.85–2.79)	1.24 ± 0.27 *
		CT	119 (43.1)	0.94 (0.65–1.89)	4.31 (3.86–4.96)	2.37 (1.85–2.86)	1.24 ± 0.30 *
		TT	41 (14.9)	0.94 (0.72–1.62)	4.15 (3.56–4.97)	2.13 (1.84–2.97)	1.04 ± 0.32 *
	Case	CC	115 (44.4)	1.98 (0.98–2.76)	4.67 (4.11–5.51)	2.64 (2.14–3.14)	1.06 ± 0.31
		CT	124 (47.9)	1.82 (1.05–2.60)	4.49 (3.98–5.63)	2.54 (2.04–3.12)	1.03 ± 0.27
		TT	20 (7.7)	1.44 (0.91–2.57)	4.48 (3.95–5.08)	2.44 (1.89–2.91)	1.03 ± 0.28
rs2230806	Control	AA	40 (14.5)	0.88 (0.66–1.46)	4.35 (3.66–4.93)	2.40 (1.90–2.92)	1.25 ± 0.25
		AG	117 (42.4)	0.90 (0.64–1.63)	4.17 (3.76–4.76)	2.27 (1.88–2.85)	1.22 ± 0.29
		GG	119 (43.1)	0.92 (0.66–1.86)	4.32 (3.67–4.96)	2.37 (1.81–2.79)	1.22 ± 0.32
	Case	AA	42 (16.2)	1.69 (1.13–2.32)	4.78 (4.31–5.16)	2.58 (2.04–2.96)	1.01 ± 0.24
		AG	128 (49.4)	2.08 (0.99–2.76)	4.69 (4.02–5.75)	2.65 (2.00–3.28)	1.04 ± 0.29
		GG	89 (34.4)	1.54 (0.92–2.55)	4.37 (3.95–5.15)	2.49 (2.06–2.90)	1.06 ± 0.31
rs1800976	Control	CC	50 (18.1)	0.91 (0.65–1.48)	4.03 (3.45–4.61)	2.19 (1.84–2.94)	1.24 ± 0.32
		CG	127 (46.0)	0.94 (0.65–1.86)	4.34 (3.97–4.81)	2.40 (1.89–2.80)	1.24 ± 0.28
		GG	99 (35.9)	0.88 (0.61–1.69)	4.23 (3.65–4.99)	2.24 (1.81–2.94)	1.19 ± 0.30
	Case	CC	49 (18.9)	1.26 (0.91–2.49) *	4.56 (3.93–5.45) *	2.45 (1.99–2.96) *	1.07 ± 0.28
		CG	124 (47.9)	2.09 (1.19–2.76) *	4.83 (4.16–5.74) *	2.70 (2.20–3.28) *	1.01 ± 0.28
		GG	86 (33.2)	1.64 (0.90–2.48) *	4.36 (3.94–5.01) *	2.48 (1.94–2.92) *	1.07 ± 0.29
rs4149313	Control	AA	86 (31.2)	0.94 (0.61–2.05)	4.30 (3.65–4.76)	2.27 (1.74–2.84)	1.17 ± 0.28 *
		AG	135 (48.9)	0.88 (0.63–1.52)	4.23 (3.75–4.88)	2.27 (1.84–2.76)	1.22 ± 0.29
		GG	55 (19.9)	0.90 (0.67–1.69)	4.38 (3.80–4.98)	2.45 (1.97–2.99)	1.31 ± 0.29 *
	Case	AA	64 (24.7)	1.81 (0.99–2.64)	4.57 (4.09–5.24)	2.64 (2.26–2.93)	1.05 ± 0.29
		AG	121 (46.7)	1.88 (1.05–2.73)	4.67 (4.12–5.52)	2.66 (2.00–3.17)	1.03 ± 0.29
		GG	74 (28.6)	1.74 (0.91–2.53)	4.45 (3.93–5.54)	2.43 (1.89–3.08)	1.06 ± 0.29

Notes: TG, triglyceride; TC, total cholesterol; LDL-C, low-density lipoprotein-cholesterol; HDL-C, high-density lipoprotein-cholesterol; * *p* < 0.05.

## 4. Discussion

The results of the present study showed that the levels of TC, TG, and LDL-C were lower in normal weight than those in overweight/obese subjects; whereas the level of HDL-C was higher. These findings were line with those of previous studies [16,17,18]. Elevated TG is one of the major characteristics of dyslipidemia in obesity, and TGs are lipolyzed in the intestinal lumen into free fatty acids (FFAs). Existing studies have found that abnormal TG and FFA metabolism might partly result from insulin resistance [19,20,21,22]. Insulin plays an important role in lipid synthesis, and liver is the main target organ, thereby possibly altering lipid metabolism and contributing to dyslipidemia [23]. Insulin resistance can also decline the activity of lipoprotein lipase. The metabolism of very-low-density lipoproteins (VLDLs) decreases, and the level of VLDL increases. Moreover, increasing the levels of TG can lead to lower levels of HDL-C [24].

Our study showed no significant difference between case group and the control group in age, and the genotypic frequency was consistent with Hardy-Weinberg Equilibrium, with group representative. The frequency distribution of the six SNPs also varied in different research groups. Our research showed that rs2515602 C allele frequency (42.1%) was higher than European (26.3%), but was lower than that in Han Chinese (73.7%) [25]. Rs3890182 G allele frequency (97.4%) was similar to that Han Chinese in Beijing (93.0%) and Japanese in Tokyo (94.8%) [26]. The frequency of rs2275542 T allele in our study populations (34.1%) was obviously lower than that in Han Chinese in Beijing (77.9%) and European (74.3%) [27]. We found that Rs2230806 A allele(38.2%) in our study was similar to Han Chinese in Beijing (41.9%) and Japanese in Tokyo (42.4%) [28]. We also found that rs1800976 C allele(41.9%) was varied in different other populations [29]. The frequency of rs4149313 G allele in our study subjects (48.0%) was higher than that in another study population [30]. These results showed significant racial/ethnic variations in allelic frequencies in the ABCA1 gene.

Serum lipid levels are affected by exogenous and endogenous factors, while increasing rates of overweight and obesity may partly be attributed to dyslipidemia [31]. The ABCA1 gene is closely related to the metabolism of lipids [3,4]. Some scholars have reported that the polymorphism of ABCA1 gene is not only associated with lipid metabolism, but also with other metabolic diseases such as type 2 diabetes [32], obesity and metabolic syndrome [33]. To date, few studies have examined the association of ABCA1 gene polymorphisms and overweight/obese. This study demonstrated that polymorphism distributions of rs2515602, rs2275542 rand rs4149313 were significantly different between the normal weight group and overweight/obese group. Single factor logistic regression was found to have a significant correlation with the risk of overweight/obesity in three loci. The results of multi factor logistic regression showed that rs2515602 and rs4149313 were still significant. Haplotype analysis with all six SNPs further supports the strong association between ABCA1 gene polymorphisms and overweight/obese in our study subjects. This association may partly result from ABCA1 gene mutations, which can lead to an inflammatory reaction and an increase in the risk of overweight/obesity [34,35,36]. LD analysis of ABCA1 gene 6 SNPs showed that there were significant LD between rs2275542 with rs3890182 and rs2515602 with rs4149313. Nearly no recombination occurred in the genetic process and as a whole to the next generation. These results suggest that there is association between ABCA1 gene polymorphism and overweight/obese.

Information about the association of ABCA1 gene haplotype and overweight/obesity is limited. In this study, we observed that C-C-C-A-G-G and T-C-G-A-G-G haplotypes were significantly more frequent in the case group than in the control group, whereas the T-T-G-G-G-A haplotypes was less frequent in the overweight/obese group than in the normal weight group. These findings suggested that the C-C-C-A-G-G and T-C-G-A-G-G haplotypes may serve as risk factors of overweight/obesity more than the C-T-G-G-G-A haplotype among Uyghurs, whereas the T-T-G-G-G-A haplotype may serve as a protective factor of overweight/obesity more than the C-T-G-G-G-A haplotype among Uyghurs.

rs2230806 and rs4149313 are located in the exon region, whereas rs2275542 and 3890182 are located in the intron region and rs1800976 is located in promoter [37]. These SNPs variants will effects ABCA1 expression or function, thereby affect serum lipids. In recent years, numerous studies have showed relationship between the ABCA1 gene polymorphism and serum lipid levels, however, still remain inconsistent in different races [10,11,38,39,40,41,42]. rs2515602 polymorphism has been found to correlate strongly with HDL-C levels in coronary artery risk development in young adults and TG levels in African Americans [11,43]. rs2275542 polymorphism was significantly associated with the HDL-C level in the Suita population [10]. rs2230806 polymorphism was significantly associated with the HDL-C level in Egyptians and Asians; rs2230806-G allele was associated with a decrease in HDL C levels in CAD patients [39,44,45]. However, another study shows that the rs2230806-G allele was associated with increase in HDL-C levels in obese people [46]. On the contrary, the association of ABCA1 rs2230806 polymorphism and HDL-C levels not observed in young Greek nurses and coronary heart disease patients [47,48]. The ABCA1 rs1800976 polymorphism was associated with the HDL-C level in the Suita population, but no association between ABCA1 rs1800976 genotype and lipid levels was found in another study [10,49]. The ABCA1 rs4149313 and rs3890182 polymorphisms were also not associated with the HDL-C level [11,41,42]. In our present study, we showed that association between ABCA1 gene polymorphisms and some plasma lipid levels. The results are in line with some previous studies which supports the association between ABCA1 gene polymorphism and serum lipid levels.

## 5. Study Limitations

Our study had several potential limitations. First, the sample size in our study was small. No individuals with the rs3890182 AA genotype were detected in our case group, and the number of subjects with rs3890182 AA genotype in control group was also small. Second, although we have discussed the relationship between ABCA1 six SNPs polymorphisms and overweight/obese, the mechanism of ABCA1 gene polymorphism and overweight/obesity remains unclear. Thus, further studies are required to understand the role of ABCA1 gene polymorphisms in developing overweight/obese. Finally, overweight/obese is affected by multiple environmental and genetic factors and their interactions. Many environmental and genetic factors and their interactions remain unclear and undetected.

## 6. Conclusions

The present study shows that there is no significant difference in the genotypic and allelic frequencies of ABCA1 rs2230806, rs3890182 and rs1800976 polymorphisms between a normal weight group and an overweight/obese group. We also found that rs2515602, rs2230806 and rs4149313 in ABCA1 gene variants were significantly related to risk of overweight/obesity. Finally, we determined that C-C-C-A-G-G and T-C-G-A-G-G haplotypes may serve as risk factors of overweight/obesity among Uyghurs, whereas the T-T-G-G-G-A haplotype may serve as a protective factor of overweight/obesity among Uyghurs. The differences in serum lipid levels between normal weight and overweight/obese subjects might partly result from ABCA1 gene variant.
